# Albuminuria Associated with Habitual Use of Opium

**Published:** 1884-12

**Authors:** F. P. Mann


### ARTICLE III.

### ALBUMINURIA ASSOCIATED WITH HABITUAL USE OF OPIUM.

*By* F. P. MANN, M. D.

The following case may prove interesting, presenting, as it does, some features not often encountered in the routine of special practice.

Mrs. K., married, aged twenty-eight. Has one child, three years of age. Menstruation ceased eighteen months ago, unattended by usual symptoms of meno-pause, and up to present time she has experienced no inconvenience from suspended menstruation. About two years ago she commenced the use of opium, and has continued it daily,in gradually increasing doses, ever since. She now takes an ounce of Tinct. Opii, or its equivalent in morphine or tinct. opii camph. during twenty-four hours. When first visited, May 22nd, evening, her condition was as follows: Body well nourished. Face full. Pupils obstinately contracted. Temp. 101¼. Pulse 115. No œdema. Constant nausea and frequent vomiting. Vomiting commenced soon after swallowing about 4 oz. of paregoric. Bowels constipated at present, though generally regular, notwithstanding the habitual use of opium. Tenderness and dull pain when deep pressure is made over space occupied by right kidney; no pain or tenderness on left side. Tongue coated. Great restlessness. Urine, spec. grav. 1020, very acid; set aside for six hours gave large deposit of urates thickly sprinkled with crystals of uric acid. Coloring matter normal; 500 grs. tested for urea showed deficiency. A few drops of nitric acid added to urine freed from deposit, threw down precipitate of albumen equal in bulk to about one-third the quantity of urine tested. Corroborated by boiling test. Microscopical examination showed renal epithelium and a few hyaline casts. Patient, though occupying a high social position, is demoralized by the use of opium. Before prescribing, I insisted that the bed should be made up, and to accomplish this, patient was placed upon a lounge. My object was to unearth any concealed bottles of laudanum, paregoric or morphine; none were found. A cataplasm of mustard and linseed, one part to two, was applied to epigastrium and covered with oiled silk, with directions to allow it to remain for twelve hours. Small pieces of ice to relieve thirst and nausea. Restlessness and desire for accustomed opiate controlled by twenty gr. doses chloral hydrate combined with potass. brom. 30 grs. in sol. every four hours until sleep was produced. Slept soundly after third dose, to be given in future only at night, and quantity gradually reduced as soon as condition of patient will admit of it. Cathartic exhibited, which moved the bowels freely. The urine was kept slightly alkaline by the following: Potass. bicarb., ℥iss.; acid benzoici, gr. i.; aq., ℥vi. Tablespoonful every four hours with one-third tumbler of water. Patient was carefully watched to prevent the possibility of procuring opium in any form. This treatment was pursued with slight variation for two weeks; at this time, albumen, which had been gradually diminishing in quantity, wholly disappeared. Care was taken before testing to render the urine slightly acid to avoid the possibility of any free alkali preventing the prompt precipitation of albumen. After twenty-one days all desire for opiates ceased, and but one dose of sedative mixture was required at night. The alkaline solution was continued for three months, and no return of albuminuria has occurred up to present time. Average specific grav., 1018 to 1020. No deposit of urates or uric acid. The sudden disappearance of albumen for days and weeks together during the progress of different forms of Bright's disease, is of common occurrence, and in the cirrhotic variety it may be absent, or present in very small quantity, as in a case which I recently attended in consultation with Dr. Austin Flint, Sen., of New York, where repeated testing failed to show more than a trace of albumen, uræmic convulsions being the first symptom that attracted attention. In this case recovery seems assured, as patient appears in perfect healthat present time.

